# Piceatannol exhibits anti‐inflammatory effects on macrophages interacting with adipocytes

**DOI:** 10.1002/fsn3.366

**Published:** 2016-05-16

**Authors:** Takayuki Yamamoto, Yongjia Li, Yuki Hanafusa, Yu‐Sheng Yeh, Hiroko Maruki‐Uchida, Shinpei Kawakami, Masahiko Sai, Tsuyoshi Goto, Tatsuhiko Ito, Teruo Kawada

**Affiliations:** ^1^ Research InstituteMorinaga and Company Ltd.2‐1‐1 ShimosueyoshiTsurumi‐kuYokohama230‐8504Japan; ^2^ Laboratory of Molecular Functions of FoodDivision of Food Science and BiotechnologyGraduate School of AgricultureKyoto UniversityUjiKyoto611‐0011Japan; ^3^ Research Unit for Physiological ChemistryCenter for the Promotion of Interdisciplinary Education and ResearchKyoto UniversityKyoto606‐8502Japan

**Keywords:** 3T3‐L1 Adipocytes, C3H10T1/2 adipocytes, inflammation, piceatannol, RAW264.7 macrophages, UCP1

## Abstract

Piceatannol (PIC), a natural analog of resveratrol (RES), is a phytochemical found in passion fruit seeds. To clarify the effects of PIC on obesity‐induced inflammation in adipose tissue, we investigated the anti‐inflammatory activity of PIC‐related compounds (PIC, RES, and metabolites from PIC) in culture models of obese adipose tissue. Lipopolysaccharide (LPS) and conditioned medium from 3T3‐L1 adipocytes (3T3‐L1‐CM) enhanced proinflammatory gene expression and synthesis of nitric oxide (NO), tumor necrosis factor‐*α* (TNF‐*α*), and interleukin‐6 (IL‐6) in RAW264.7 macrophages. Although each compound inhibited the mRNA expression of iNOS (inducible NO synthase), TNF‐*α*, and IL‐6, PIC potently inhibited them, and 30 *μ*mol/L PIC suppressed the LPS‐ and 3T3‐L1‐CM‐induced mRNA expression of iNOS (70.4% and 69.2% suppression, respectively), TNF‐*α* (42.6% and 47.0% suppression), and IL‐6 (27.3% and 42.1% suppression). PIC also significantly suppressed production of NO (80.3% suppression) and inflammatory cytokines (TNF‐*α*; 33.7% suppression, IL‐6; 66.5% suppression). Furthermore, PIC was found to rescue the uncoupling protein 1 mRNA expression induced by isoproterenol in 10T1/2 adipocytes, which was suppressed by LPS‐activated macrophages. These results suggest that PIC may attenuate the pathologic inflammation triggered by adipose tissues.

## Introduction

Obesity is characterized by excessive accumulation of white adipose tissue (WAT). This tissue is composed of various cell types, including mature adipocytes, preadipocytes, fibroblasts, endothelial cells, and macrophages. Recent studies have revealed that low‐grade chronic inflammation caused by macrophages interacting with hypertrophied adipocytes can lead to metabolic syndrome (i.e., cardiovascular disease, insulin resistance, type 2 diabetes, and hypertension) (Fernández‐Real and Ricart 2003; Dandona et al. 2004). This interaction is believed to occur via the release of monocyte chemoattractant protein‐1 by hypertrophied adipocytes, which leads to the infiltration of macrophages into adipose tissue (Kanda et al. 2006). These adipocytes also secrete free fatty acid (FFA), which activates the infiltrating macrophages and stimulates their secretion of tumor necrosis factor‐*α* (TNF‐*α*) to induce insulin resistance and lipolysis in WAT (Hotamisligil et al. 1993, 1994; Yu et al. 2006). The concentration of FFA is increased upon lipolysis, which further activates the infiltrating macrophages and results in the overproduction of TNF‐*α* (Suganami et al. 2005). Thus, inhibition of the chronic inflammation within WAT is an important therapeutic strategy in the treatment of insulin resistance.

Brown adipose tissue (BAT) is a highly thermogenic tissue that dissipates energy as heat by using uncoupling protein 1 (UCP1) to mediate the uncoupling of oxidative phosphorylation from ATP synthesis (Rousset et al. 2004). As such, BAT was long believed to be present in humans primarily during the neonatal period. However, modern positron emission tomography (PET) scanning methods have been used to detect significant deposits of UCP1‐expressing adipocytes in adults (Cypess et al. 2009; Saito et al. 2009; Virtanen et al. 2009). It has also been reported that brown‐like adipocytes are induced in WAT depots by various stimuli to trigger mitochondrial respiration and energy expenditure similar to that of BAT (Wu et al. 2012). This browning of WAT is therefore expected to occur in disease states that involve excess adipose tissue, including obesity and diabetes. Interestingly, inflammation induced by macrophages is known to suppress UCP1 mRNA induction by isoproterenol in C3H10T1/2 adipocytes, a commonly used model of brown‐like adipocytes (Sakamoto et al. 2013). Thus, macrophage‐mediated inflammation can promote pathologic changes in WAT, and also influence the heat production of brown‐like adipocytes.

Piceatannol (PIC), a structurally related analog of resveratrol (RES), is a naturally occurring stilbene derivative present in high concentration in passion fruit (*Passiflora edulis*) seeds (Matsui et al. 2010). Both RES and PIC are known to activate sirtuin 1 (SIRT1), a NAD^+^‐dependent deacetylase linked to the regulation of life span and caloric restriction (Kahyo et al. 2008; Baur 2010). We have previously reported that PIC displays a variety of biological activities, such as skin protection (Matsui et al. 2010; Maruki‐Uchida et al. 2013), vasodilatation (Sano et al. 2011; Kinoshita et al. 2013), and lowering of blood glucose levels (Uchida‐Maruki et al. 2015). Furthermore, we used an in vivo (rat) model to demonstrate that the oral absorption of PIC is superior to that of RES, and that methylated metabolites, including isorhapontigenin (IRP), were detected in the plasma of animals dosed with PIC, but not those dosed with RES (Setoguchi et al. 2014). We have recently found that PIC and its metabolites upregulate the expression levels of SIRT1 mRNA using a THP‐1 human monocytic cell line (Kawakami et al. 2014).

While both PIC and RES are known to exhibit anti‐inflammatory properties, their pharmacologic profiles display variation. For example, both of these agents suppress the activation and nuclear translocation of nuclear factor‐κB (NF‐*κ*B), a central transcriptional regulator in inflammatory responses (Djoko et al. 2007), but only RES analogs (due to their hydroxyl groups) are able to suppress TNF‐*α* in RAW264.7 macrophages (Son et al. 2014). Furthermore, pterostilbene, a dimethylated derivative of RES, inhibits the inflammatory interaction between 3T3‐L1 adipocytes and RAW264.7 macrophages (Hsu et al. 2013). Despite much study on the effects of PIC and RES against fulminant lipopolysaccharide (LPS) stimulation, PIC has not been verified to diminish chronic inflammation resulting from the interaction between adipocytes and macrophages. Furthermore, whereas various food compounds have been shown to possess anti‐inflammatory properties in culture models mimicking obese adipose tissues, there are few reports on anti‐inflammatory food compounds that ameliorate inflammation‐induced adipocyte dysfunction, including suppression the induction of UCP1 mRNA expression. In this study, we determined and compared the anti‐inflammatory effects of PIC to those of its known metabolites, using LPS‐stimulated macrophages. We also investigated the effects of PIC and RES on the TNF‐*α*/FFA‐mediated inflammatory cycle between adipocytes and macrophages, and on the browning of white adipocytes.

## Materials and Methods

### Chemicals

**Figure 1:**
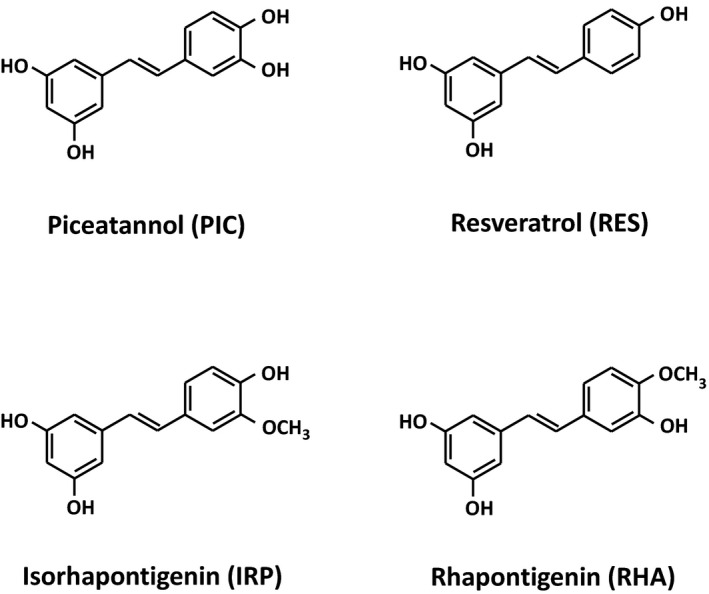
The chemical structures of stilbene derivatives: piceatannol (PIC), resveratrol (RES), isorhapontigenin (IRP), and rhapontigenin (RHA).

PIC, RES, IRP, and rhapontigenin (RHA) were purchased from Tokyo Chemical Industry Co., Ltd (Tokyo, Japan). Figure 1 displays the chemical structures of all stilbenes used in this study. Insulin and dexamethasone were purchased from Wako Pure Chemical Industries, Ltd. (Osaka, Japan). 3‐Isobutyl‐1‐methylxanthine, troglitazone, LPS from *Escherichia coli*, serotype 055:B5, and (−)‐isoproterenol hydrochloride were purchased from Sigma (St. Louis, MO). Fetal bovine serum (FBS) was obtained from Hyclone (Logan, UT). All other materials used in the cellular experiments were purchased from Gibco BRL (New York, NY).

### Cell culture

3T3‐L1 preadipocytes (IFO 50416, JCRB Cell Bank, Osaka, Japan) were cultured in Dulbecco's modified Eagle's medium (DMEM) containing 10% FBS, penicillin (100 U/mL), and streptomycin (100 *μ*g/mL) at 37°C in a humidified atmosphere of 5% CO_2_. Differentiation of 3T3‐L1 preadipocytes was induced by treatment with adipogenic agents (0.5 mmol/L 3‐isobutyl‐1‐methylxanthine, 0.25 *μ*mol/L dexamethasone, 10 *μ*g/mL insulin, and 1 *μ*mol/L troglitazone) in DMEM containing 10% FBS for 2 days after the cells reached confluence (day 0), as previously described (Hirai et al. 2007). The medium was then replaced with fresh DMEM containing 10% FBS and insulin (5 *μ*g/mL), and again every 2 days. After 20 days, the cells that accumulated large lipid droplets were used as hypertrophied 3T3‐L1 adipocytes. The serum‐free medium of these hypertrophied adipocytes was cultured for 12 h, collected as 3T3‐L1 conditioned medium (3T3‐L1‐CM), and stored at −20°C until use. The 3T3‐L1 preadipocytes without lipid droplets 3 days post induction of differentiation were used as control cells.

RAW264.7 macrophages (Public Health England, catalog number 91062702, Dainippon Sumitomo Pharma, Osaka, Japan) were cultured in DMEM containing 10% FBS, penicillin (100 U/mL), and streptomycin (100 *μ*g/mL) at 37°C in a humidified atmosphere of 5% CO_2_, as previously described (Lin et al. 2013). The cells were seeded in 12‐well plates (2.4 × 10^5^ cells/well) and treated with either LPS (30 ng/mL) or 3T3‐L1‐CM. Each stilbene compound was administrated simultaneously with LPS or 3T3‐L1‐CM at various concentrations. At 4 and 24 h post treatment, both RNA and culture supernatants were collected and stored at −20°C until quantification of gene expression and measurement of TNF‐*α*, interleukin‐6 (IL‐6), and nitric oxide (NO) concentrations, respectively, were undertaken. RAW264.7 macrophages were cultured in 10 mm dishes; some were stimulated with LPS (30 ng/mL) for 12 h, others were not. Each stilbene compound was administered, at various concentrations, simultaneously with LPS. The cells were then cultured in serum‐free DMEM for 12 h, the resulting RAW264.7 CM (RAW‐CM) was collected, and stored at −20°C until use.

C3H10T1/2 preadipocytes (10T1/2) (JCRB 9080, JCRB Cell Bank) were cultured and differentiation induced using the same methods described above for the 3T3‐L1 preadipocytes. The medium was then replaced with fresh DMEM containing 10% FBS and insulin (5 *μ*g/mL), and again every 2 days. Eight days after the induction of differentiation, the medium was replaced with RAW‐CM diluted with an equal volume of DMEM and pretreated for 12 h. The cells were then cultured with or without isoproterenol (10 *μ*mol/L) for 8 h, as previously described (Sakamoto et al. 2013).

For all experiments, the stilbene compounds were administered in concentrations that had no significant effect on the viability of the cell lines used.

### RNA preparation and real‐time PCR

Determination of mRNA expression was performed as previously described (Kawakami et al. 2014). Total RNA samples extracted from cells were reverse transcribed into complementary DNA, and real‐time polymerase chain reaction (PCR) were performed in triplicate using a Light Cycler 480 Real‐Time PCR system II (Roche Diagnostics, Mannheim, Germany). The PCR primers that were used are listed in Table S1. The amplification conditions were as follows: 50°C for 2 min, then 95°C for 10 min, followed by 45 cycles at 95°C for 10 sec, and then 60°C for 25 sec. To compare mRNA expression levels among samples, copy numbers of all transcripts were divided by that of mouse GAPDH and 36B4 showing a constant expression level in macrophages and adipocytes, respectively. All mRNA expression levels are represented as ratios relative to the control in each experiment.

### NO assay

The amount of nitrite present in the cell‐free culture supernatants was measured using the Griess reagent (Granger et al. 1996). Briefly, 100 *μ*L of supernatant was mixed with an equal volume of Griess reagent (0.1% *N*‐1‐naphthyl‐ethylenediamine in distilled water and 1% sulfanilamide in 5% phosphoric acid, 1:1) in a 96‐well flat‐bottom plate. After 10 min, the absorbance at 570 nm was measured, and the amount of nitrite present was calculated from the NaNO_2_ standard curve.

### Measurement of cytokine production

The concentrations of TNF‐*α* and IL‐6 in the culture supernatants were determined using Mouse TNF‐*α* and IL‐6 READY‐SET‐GO! kits (eBioscience, San Diego, CA) in accordance with the manufacturer's instructions.

### Statistical analysis

Data are presented as means ± standard error of the mean. Statistical evaluations were performed via one‐way analysis of variance (ANOVA), followed by the Dunnett test for multiple comparisons using SPSS software (SPSS Inc., Tokyo, Japan). A value of *P *<* *0.05 was considered significant.

## Results

### Effect of stilbenes on the expression of genes related to inflammation in LPS‐induced RAW264.7 macrophages

To examine the effect of each stilbene (PIC, RES, IRP, and RHA) on macrophage activation, we measured the expression of genes related to inflammation in RAW264.7 macrophages.

**Figure 2:**
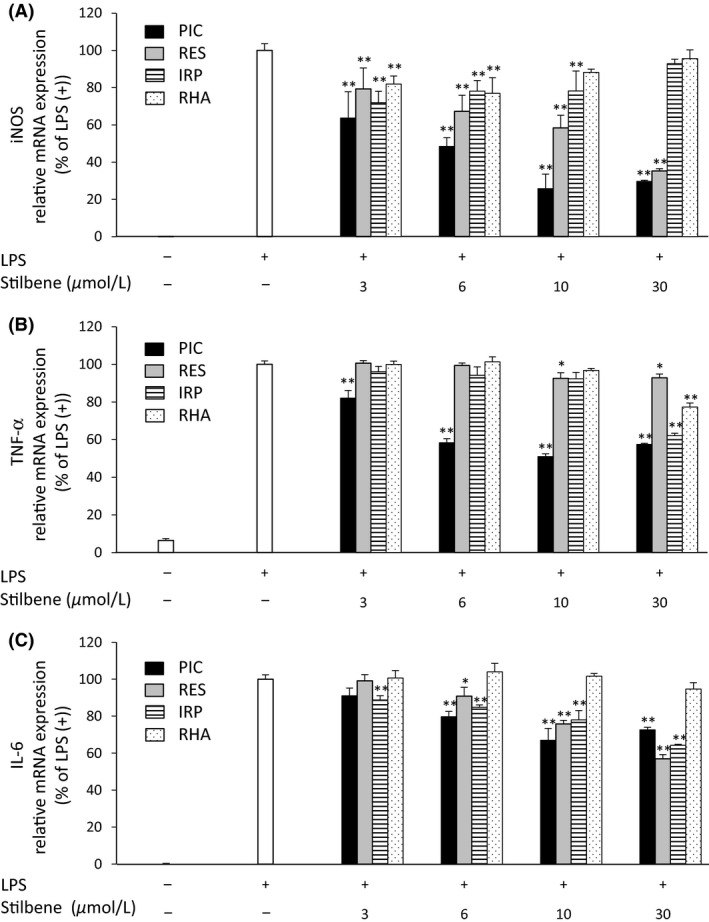
The effects of stilbenes (PIC, RES, IRP, and RHA) on mRNA expression of iNOS (A), TNF‐*α* (B), and IL‐6 (C) in RAW264.7 macrophages activated by LPS. RAW264.7 macrophages were incubated in the absence or presence of stilbene (at the indicated concentrations) with LPS (30 ng/mL) for 4 h. mRNA expression was then determined by RT‐PCR. The ratio of proinflammatory‐related genes to *GAPDH* was determined and expressed as % of LPS (+). Values are means ± SEM for 4–6 samples. *P <* *0.05, **P <* *0.01 compared with LPS alone. PIC, piceatannol; RES, resveratrol; IRP, isorhapontigenin; RHA, rhapontigenin; iNOS, inducible nitric oxide synthase; TNF‐*α*, tumor necrosis factor‐*α*; IL‐6, interleukin‐6; LPS, lipopolysaccharide; RT‐PCR, real‐time polymerase chain reaction.

As shown in Figure 2, LPS (30 ng/mL) stimulation significantly increased the expression of inducible NO synthase (iNOS) (Fig. 2A), TNF‐*α* (Fig. 2B), and IL‐6 (Fig. 2C) genes in RAW264.7 macrophages. PIC significantly suppressed the LPS‐induced expression of these genes in a dose‐dependent manner, ranging from 3 to 30 *μ*mol/L. The relative expression levels of iNOS, TNF‐*α*, and IL‐6 were 29.6%, 57.4%, and 72.7%, respectively, at the 30 *μ*mol/L PIC dosage. The overall anti‐inflammatory profile of PIC was more pronounced than RES, IRP, and RHA in regard to their inhibition of key gene expressions in stimulated RAW264.7 macrophages.

### Effect of stilbenes on NO production in LPS‐induced RAW264.7 macrophages

**Figure 3:**
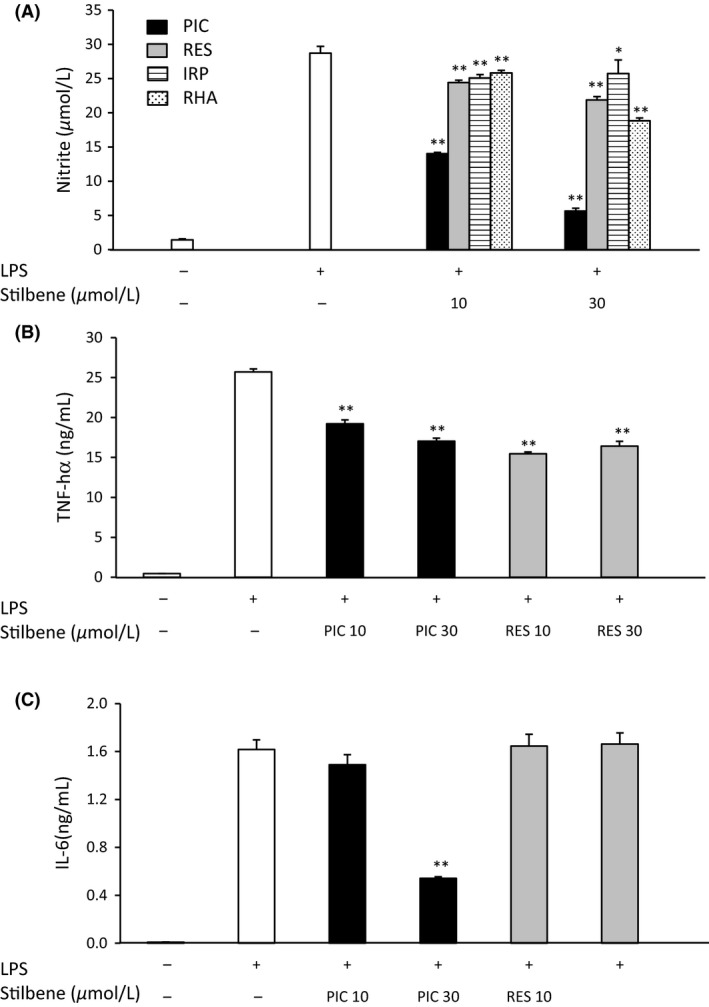
The effects of stilbenes (PIC, RES, IRP, and RHA) on NO production (A), and PIC and RES on the secretion of TNF‐*α* (B) and IL‐6 (C) in RAW264.7 macrophages activated by LPS. RAW264.7 macrophages were incubated in the absence or presence of stilbene (at the indicated concentrations) with LPS (30 ng/mL) for 24 h. The amounts of NO and cytokines in the culture medium were measured using the Griess method and ELISA, respectively. Values are means ± SEM for 4 samples. *P <* *0.05, **P <* *0.01 compared with LPS alone. PIC, piceatannol; RES, resveratrol; IRP, isorhapontigenin; RHA, rhapontigenin; NO, nitric oxide; TNF‐*α*, tumor necrosis factor‐*α*; IL‐6, interleukin‐6; LPS, lipopolysaccharide.

Since the gene expression of iNOS was found to be suppressed by stilbenes, we examined the NO production in LPS‐induced RAW264.7 macrophages. NO production was significantly increased (from 1.4 to 28.7 *μ*mol/L) in RAW264.7 macrophages stimulated with LPS for 24 h. However, administration of PIC (10 and 30 *μ*mol/L) to these cells resulted in significant suppression of LPS‐induced NO production (51.1% and 80.3% suppression, respectively) (Fig. 3A). Although the other stilbenes evaluated also suppressed NO production in these cells (RES; 23.8%, IRP; 10.3%, RHA; 34.4% suppression, at 30 *μ*mol/L each), PIC clearly exhibited the most dramatic effect.

### Effect of PIC and RES on the production of inflammatory cytokines in LPS‐induced RAW264.7 macrophages

RAW264.7 macrophages stimulated with LPS were demonstrated to produce significantly increased amounts of TNF‐*α* and IL‐6 compared to unstimulated cells. Both PIC and RES significantly reduced the production of TNF‐*α* in LPS‐induced cells to nearly the same degree (Fig. 3B). PIC (30 *μ*mol/L) reduced the IL‐6 production in LPS‐stimulated macrophages to 33.5% of the amount produced in the untreated control cells. However, RES did not suppress IL‐6 secretion in LPS‐induced RAW264.7 cells (Fig. 3C).

### Effect of PIC and RES on the expression of genes related to inflammation in 3T3‐L1‐CM‐induced RAW264.7 macrophages

The CM collected from hypertrophied 3T3‐L1 adipocytes was used to stimulate activated RAW264.7 macrophages in order to more accurately model the chronic inflammation within adipose tissue. Thus, the anti‐inflammatory effect(s) of PIC and RES were evaluated in this system.

**Figure 4:**
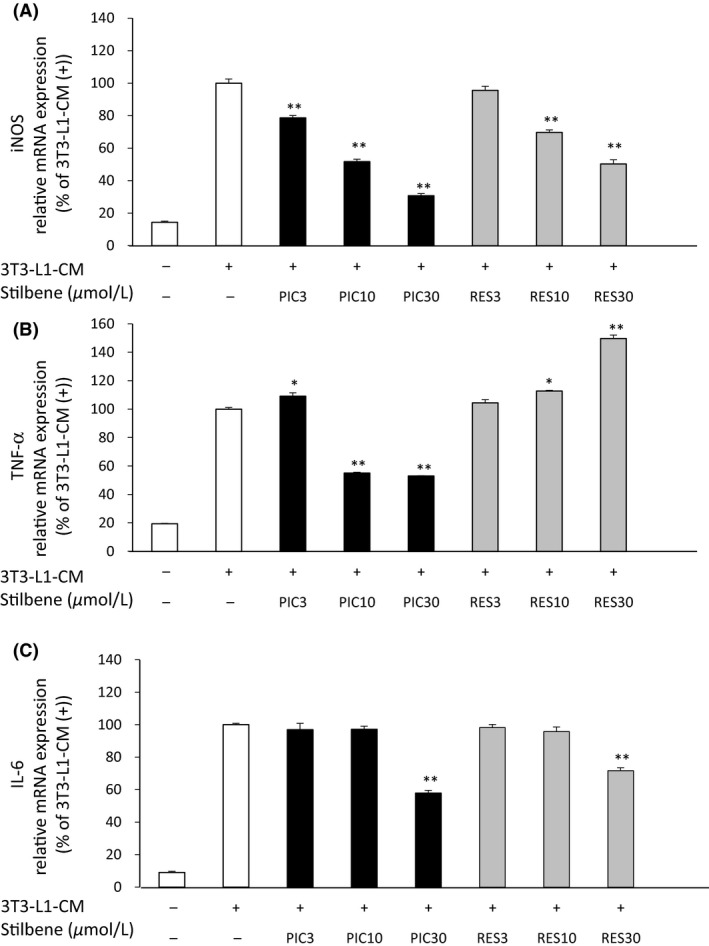
The effects of PIC and RES on mRNA expression of iNOS (A), TNF‐*α* (B), and IL‐6 (C) in RAW264.7 macrophages activated by CM derived from hypertrophied 3T3‐L1 adipocytes (3T3‐L1‐CM). RAW264.7 macrophages were incubated in the absence or presence of PIC or RES (at the indicated concentrations) with CM obtained from fully differentiated (day 20) 3T3‐L1 adipocytes for 4 h. The medium derived from early differentiated (day 3) 3T3‐L1 adipocytes (indicated as 3T3‐L1‐CM (−) in the figure) was used as the control. mRNA expression levels were determined by RT‐PCR. Values are means ± SEM for four samples. *P <* *0.05, **P <* *0.01 compared with the 3T3‐L1‐CM (+) only. PIC, piceatannol; RES, resveratrol; iNOS, inducible nitric oxide synthase; TNF‐*α*, tumor necrosis factor‐*α*; IL‐6, interleukin‐6; CM, conditioned medium; RT‐PCR, real‐time polymerase chain reaction.

Stimulation with 3T3‐L1‐CM significantly increased the expression of iNOS, IL‐6, and TNF‐*α* genes in RAW264.7 macrophages (Fig. 4). PIC and RES were both found to significantly suppress iNOS mRNA expression in a dose‐dependent manner (Fig. 4A). These two agents also suppressed IL‐6 mRNA expression, but only at the highest (30 *μ*mol/L) concentration evaluated (Fig. 4C). Only PIC (at 10 and 30 *μ*mol/L concentrations) was able to inhibit TNF‐*α* expression in 3T3‐L1‐CM‐induced macrophages.

### Effect of PIC and RES on RAW‐CM‐induced UCP1 expression in C3H10T1/2 adipocytes

**Figure 5:**
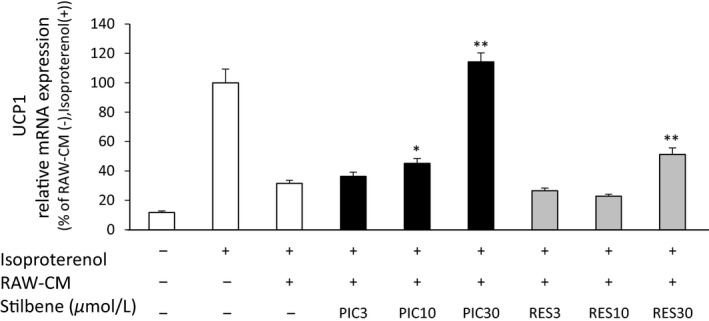
The effects of PIC and RES on UCP1 mRNA expression in C3H10T1/2 (10T1/2) adipocytes. 10T1/2 adipocytes were incubated with CM obtained from either nonactivated macrophages or LPS‐activated macrophages in the absence or presence of PIC or RES (at the indicated concentrations) for 12 h before isoproterenol (10 *μ*mol/L) treatment for 8 h. The medium obtained from nonactivated macrophages (indicated RAW‐CM(−) in the figure) was used as the control. UCP1 mRNA expression levels were determined by RT‐PCR. The ratio of *UCP1* to *36B4* was determined and expressed as % of LPS (−) and isoproterenol (+). Values are means ± SEM for six samples. *P <* *0.05, **P <* *0.01 compared with the RAW‐CM (+) and isoproterenol (+). PIC, piceatannol; RES, resveratrol; UCP1, uncoupling protein 1; CM, conditioned medium; LPS, lipopolysaccharide; RT‐PCR, real‐time polymerase chain reaction.

As previously reported (Sakamoto et al. 2013), isoproterenol triggers an increase in the mRNA expression levels of UCP1 in 10T1/2 adipocytes, and RAW‐CM inhibits this expression by 69%. Treatment batches of RAW‐CM, containing PIC or RES (at 3, 10, and 30 *μ*mol/L), were used to evaluate the abilities of these stilbene compounds to rescue the rise in UCP1 expression levels induced by isoproterenol in 10T1/2 adipocytes. Both PIC and RES were found to rescue UCP1 mRNA expression in these adipocytes in a dose‐dependent manner (Fig. 5). PIC was more potent than RES in regard to this effect; PIC at 10 *μ*mol/L displayed nearly the same degree of recovery as RES at 30 *μ*mol/L. The induction of UCP1 mRNA expression by isoproterenol was restored to near control levels in the 30 *μ*mol/L PIC RAW‐CM treatment group.

## Discussion

PIC, an abundant component of passion fruit seeds, has been found to exhibit variety of biological activities (Hanna et al. 2012). In this study, we investigated its effects on the inflammatory changes that result from the interaction of activated macrophages with two types of adipocytes.

The anti‐inflammatory effects of PIC and RES on LPS‐activated RAW264.7 macrophages have been well studied, and differential pharmacologic profiles for these two agents are known (Islam et al. 2004; Son et al. 2014). It is, however, unknown whether PIC exhibits these same beneficial effects in models of chronic inflammation stemming from adipocytes. Our current data have demonstrated that the mRNA expression of proinflammatory mediators in RAW264.7 macrophages were induced by CM of hypertrophied 3T3‐L1 adipocytes, and that PIC significantly suppressed this phenomenon (Fig. 4). PIC has also been reported to inhibit adipogenesis of 3T3‐L1 preadipocytes via modulation of mitotic clonal expansion (Kwon et al. 2012). Collectively, these findings suggest that PIC can potentially suppress the TNF‐*α*/FFA‐mediated cycle of chronic inflammation in white adipocytes.

UCP1 is present in the mitochondrial inner membrane of brown/brown‐like adipocytes, and controls thermogenic activity (Rothwell and Stock 1979). UCP1 activity is expected to be a target for the treatment of obesity and insulin resistance (Poher et al. 2015). It has recently been described that inflammation induced by macrophages suppresses UCP1 mRNA induction in adipocytes (Sakamoto et al. 2013). This study is the first to investigate the effect of food components on UCP1 expression in adipocytes subjected to inflammatory conditions. We determined that PIC and RES both inhibit the suppression of UCP1 mRNA induction by LPS‐activated RAW‐CM in 10T1/2 adipocytes, albeit to varying degrees (Fig. 5). LPS‐activated RAW‐CM and TNF‐*α* have previously been found to suppress the induction of UCP1 mRNA in 10T1/2 adipocytes through extracellular signal‐regulated kinase activation and subsequent blockade of nuclear signaling (Sakamoto et al. 2013). Because neutralizing antibodies against TNF‐*α* partially suppressed the inhibitory effects of LPS‐activated RAW‐CM on UCP1 mRNA induction, TNF‐*α* is most likely responsible, at least in part, for the inhibitory effects of LPS‐activated RAW‐CM on UCP1 mRNA induction. Although PIC and RES both displayed similar inhibition of TNF‐*α* production (Fig. 3A), LPS‐activated RAW‐CM treated with PIC, as opposed to RES, also effectively recovered the reduced UCP1 mRNA expression in 10T1/2 adipocytes (Fig. 5). These data suggest that inflammatory mediators other than TNF‐*α* contribute to the suppression of UCP1 mRNA expression in 10T1/2 adipocytes.

In regard to its mechanism of action, PIC has been demonstrated to suppress the activation of NF‐*κ*B, as well as inhibit TNF‐induced I*κ*Ba phosphorylation, p65 phosphorylation, p65 nuclear translocation, and I*κ*Ba kinase activation in human myeloid cells stimulated with LPS (Ashikawa et al. 2002). Moreover, SIRT1 has been reported to interfere with the NF‐*κ*B signaling pathway, and thus exhibit an anti‐inflammatory function. The negative regulation of inflammation by SIRT1 (Xie et al. 2013), as well as its roles in lipid metabolism and obesity, have been well characterized (Schug and Li 2011). In endothelial cells and macrophages, SIRT1 is known to deacetylate the p65 subunit of NF‐*κ*B, thereby downregulating the expression of various proinflammatory cytokines (Stein and Matter 2011). Our previous findings have clarified that PIC, IRP, and RHA each upregulate the expression levels of SIRT1 mRNA and protein in the THP‐1 human monocytic cell line (Kawakami et al. 2014). Thus, the anti‐inflammatory effects displayed by PIC and its metabolites in this study could result from their direct suppression of NF‐*κ*B activation, and/or via activation of SIRT1.

In this study, the anti‐inflammatory effects of PIC and its metabolites (RHA and IRP) on LPS‐activated RAW264.7 macrophages were determined and compared to those of RES. The oral absorption of PIC, in its parent form, is known to be greater than that of RES. Furthermore, *O*‐methyl PIC, IRP, and RHA, all known metabolites of PIC, have been detected in the plasma of rats following its oral administration. These metabolites were not detected upon administration with RES (Setoguchi et al. 2014). Herein, IRP, RHA, and PIC all suppressed the gene expression of LPS‐induced TNF‐*α* more so than RES (Fig. 2B). These findings are consistent with the previous report that PIC exhibits a greater inhibitory potency for TNF‐*α* mRNA expression than RES (Son et al. 2014). Although IRP and RHA did not suppress LPS‐induced iNOS mRNA expression, IRP suppressed the production of IL‐6 and TNF‐*α*, and RHA suppressed the production of TNF‐*α*, albeit less potently than PIC. In regard to NO, there is no correlation between LPS‐induced mRNA expression of iNOS and NO production. Inhibition of NO production is believed to occur primarily via posttranslational mechanisms (Djoko et al. 2007). In this study, the differences between inhibitory effects on LPS‐induced NO production and mRNA expression of iNOS displayed by each stilbene may stem from variance in their individual abilities to directly scavenge intracellular NO, which RES is known to do (Lorenz et al. 2003). Ultimately, the overall anti‐inflammatory activities for each stilbene derivative were found to vary in this study. However, these effects may be enhanced for PIC in vivo, since it is well‐absorbed and transformed into active metabolites.

In conclusion, we have demonstrated that PIC, and its metabolites, exhibit beneficial anti‐inflammatory effects in a macrophage/adipocyte model of inflammation, and improve the inflammation‐induced adipocyte dysfunction such as suppression of UCP1 induction. While various food components have been shown to improve inflammatory responses between WAT and macrophages (Ando et al. 2009; Hirai et al. 2010; Kim et al. 2015), this report represents the first investigation into the anti‐inflammatory properties of PIC using two kinds of adipocytes and macrophages. The results herein suggest that PIC and its metabolites may be valuable phytochemicals for improving obesity‐related adipocyte dysfunction, and attenuating inflammation in these patients.

## Conflict of Interest

None declared.

## Supporting information


**Table S1.** Sequences of the primer sets used for real‐time PCR.
